# A Case of Accidental Vaccinia Resulting in Death

**Published:** 1887-09

**Authors:** 


A Case of Accidental Vaccinia Resulting in Death.—By Melville Jay, M. R. C. S., L. R. C. P., North Adelaide (*Australian Medical Gazette*, January, 1887).—A child, aged eight months, was the subject of eczema of the face, neck and chest. The sister of this child, aged two and a-half years, was vaccinated in the ordinary way, the vesicles going through the usual course. In about three weeks afterwards a vesicular eruption appeared on the infant, and was accompanied with feverishness, restlessness, etc. In about six days later the eruption presented all the characteristics of confluent small-pox. It was clear that the raw surface of the eczema had become infected by the virus of the vaccine pock on the elder child's arm, as the two children had slept in the same bed. The case went on well up to the fourteenth day, when she refused food, and symptoms of collapse presented themselves, and the child gradually sank, and died on the following day. The mother, who had not been vaccinated since infancy, developed several well-marked vesicles on both nipples and breast, neck and cheek. These ran through the typical course of vaccinia.

